# A 10-year retrospective analysis of hospital admissions and length of stay among a cohort of homeless adults in Vancouver, Canada

**DOI:** 10.1186/s12913-016-1316-7

**Published:** 2016-02-17

**Authors:** Angela Russolillo, Akm Moniruzzaman, Milad Parpouchi, Lauren B. Currie, Julian M. Somers

**Affiliations:** Faculty of Health Sciences, Simon Fraser University, Simon Fraser University 8888 University Drive, Burnaby, BC V5A 1S6 Canada

**Keywords:** Homelessness, Health services, Mental disorders, Hospital admission

## Abstract

**Background:**

Homelessness is associated with a very high prevalence of substance use and mental disorders and elevated levels of acute health service use. Among the homeless, little is known regarding the relative impact of specific mental disorders on healthcare utilization. The aim of the present study was to examine the association between different categories of diagnosed mental disorders with hospital admission and length of stay (LOS) in a cohort of homeless adults in Vancouver, Canada.

**Methods:**

Participants were recruited as part of an experimental trial in which participants met criteria for both homelessness and mental illness. Administrative data were obtained (with separate consent) including comprehensive records of acute hospitalizations during the 10 years prior to recruitment and while participants where experiencing homelessness. Generalized Estimating Equations were used to estimate the associations between outcome variables (acute hospital admissions and LOS) and predictor variables (specific disorders).

**Results:**

Among the eligible sample (*n* = 433) 80 % were hospitalized, with an average of 6.0 hospital admissions and 71.4 days per person during the 10-year observation period. Of a combined total 2601 admissions to hospital, 1982 were psychiatric and 619 were non-psychiatric. Significant (*p* <0.001) independent predictors of hospital admission and LOS included a diagnosis of schizophrenia or bipolar disorder, as well as high (≥32 service contacts) non-psychiatric medical service use in the community.

**Conclusions:**

Our results demonstrate that specific mental disorders alongside high non-psychiatric service use were significantly associated with hospital admission and LOS. These findings suggest the importance of screening within the homeless population to identify individuals who may be at risk for acute illness and the implementation of services to promote recovery and prevent repeated hospitalization.

**Trial Registration:**

ISRCTN57595077; ISRCTN66721740

## Background

Homeless individuals exhibit increased rates of acute health service use, including both emergency department [1–3] and hospital admissions [3–6], when compared to the general population and low socioeconomic groups [7]. The frequency and duration of hospital inpatient visits by homeless people reflects the high prevalence of acute and chronic physical illness [8–10], mental disorders and substance use [11], and mortality [12, 13] within this sub-population. Moreover, “competing priorities” [14] and the fulfillment of basic physical needs [15] inherent to surviving on the streets may serve as impediments to receiving community-based care. In addition, fragmentation of health services, un-met health care needs [2, 3, 16], and barriers to care [17, 18], may increase reliance on hospital and acute care, often for medical conditions that could be prevented or treated in community settings [4, 19].

Inpatient hospitalization rates for homeless adults are two and a half [20] to five times higher [5] in comparison to non-homeless groups, with substance use [21, 22], mental disorders [23, 24], and co-occurring disorders [6, 7], contributing to higher admission rates. Moreover, homeless patients experience longer lengths of stay [4], and higher costs per discharge when compared to housed individuals [25]. In 2011, Hwang et al. [25] found that the direct and indirect hospital costs associated with homeless people were $2559 greater than those of housed patients. These additional economic pressures, in conjunction with lengths of stay between two [25] and 4 days [4] greater after adjusting for complexity, places increased resource and financial demands on acute health services. In addition to high rates of index admissions, re-admission rates are higher within homeless populations [26, 27]. Doran et al. [27] found that of homeless adults discharged from an urban hospital, over half were readmitted within one week, often for a similar condition/diagnoses for which they had been discharged.

Although prior research has investigated reasons for admission and rates of hospitalization among homeless populations, previous studies have relied on self-report and/or survey data [3, 20, 22, 28] and are often limited to single care sites [7, 29], minimizing the ability to examine clinically accurate diagnostic data across multiple acute health care settings. Of the studies that incorporated administrative data across various care sites, analyses were restricted to time periods of five years or less [4, 5, 23], with the exception of one study with results spanning a six-year period [21].

Despite clearly documented associations between common correlates of homeless individuals (e.g., mental disorder, substance misuse, prior acute health services use) and hospitalizations [3, 28], less is known about which diagnoses lead to hospitalization. Martell et al. [5], examined a homeless cohort in Hawaii, and found an association between schizophrenia and schizoaffective disorder and longer hospitalization periods, in comparison to other mental disorders. Gelberg et al. [22] reported substantial differences in hospitalization rates between substance using and non-substance using homeless women in Los Angeles. However, there is limited research describing homeless adults’ hospitalization patterns alongside more detailed diagnostic information and spanning multi-year periods of observation. This information is crucial to enable the identification of individuals who are at risk of medical emergency, and implementation of services (e.g., supported housing) to prevent hospitalization. In order to help address this gap, the present study aimed to measure the associations between specific mental disorders and hospital service use (number of admissions and LOS) in a cohort of homeless and severely mentally ill individuals in Vancouver Canada, using province-wide administrative data spanning a 10-year period.

## Methods

### Participants

The study underwent institutional review and was approved by the Research Ethics Boards at Simon Fraser University and the University of British Columbia. Participants (*n* = 497) comprised the cohort enrolled in the Vancouver at Home Study (Trial registration: Current Controlled Trials: ISRCTN57595077 (Vancouver at Home Study: Housing First plus Assertive Community Treatment versus congregate housing plus supports versus treatment as usual) and ISRCTN66721740 (Vancouver at Home study: Housing First plus Intensive Case management versus treatment as usual).

The present study exclusively examined data collected during the pre-recruitment period (i.e. prior to randomization). Eligibility was based on: legal adult status (19 years or older), housing status (absolutely homeless or precariously housed), and presence of a mental disorder as defined by the Mini International Neuropsychiatric Interview (MINI 6.0) [30, 31]. Mental disorder status was confirmed through written diagnosis from physicians or other service providers wherever possible.

Participants were recruited between October 2009 and April 2011 based on referral from 40 community agencies serving homeless adults in Vancouver [32]. Eligibility criteria were screened by telephone and a subsequent in-person interview was conducted to formally assess eligibility and to obtain written informed consent. Development of the informed consent protocol was preceded by field-testing, including cognitive interviewing to ensure participant comprehension [33]. Interviews were discontinued if participants’ mental status was compromised by apparent acute symptoms or substance use. If eligible and willing to participate, participants completed a series of detailed interviewer administered baseline questionnaires (see Baseline Interview Data for details). Participants were presented with the opportunity to provide separate consent for researchers to receive administrative data including health service utilization. Previous publications provide details on the study objectives and methodology following randomization [31, 32]. Vancouver was successful in meeting sample size targets (*n* = 497) as determined by the study protocol [31, 32].

### Variables of interest

#### Administrative data

The present analysis examined administrative data from the Ministry of Health in the province of British Columbia (BC), Canada. Residents of BC are required to enroll under the Medical Services Plan (MSP) which provides coverage for medically necessary services as delivered by physicians and surgeons, including laboratory and diagnostic procedures. Hospital admissions and MSP services are reported to the Ministry of Health, along with diagnostic information pertaining to individual health services contacts, including acute and outpatient visits. We analyzed data for consenting participants over a 10-year period before enrolment into the trial. Dates ranged based on entry into the study (2009–2011), dating back to 1999–2001. Our dependent variables were number of acute hospital admissions and LOS in all facilities of BC measured at each pre-recruitment year during the 10-year observation period. MSP records [based on the International Classification of Diseases (ICD) -9] were examined for diagnoses of mental disorders administered by physicians during the observation period. All disorders were included within the ICD range of 290–319 (mental disorders). Substance use disorders (SUD) were identified using the three-digit codes of 291, 292, 303, 304, and 305. Non substance-related mental disorders (NSMD) consisted of all other codes within the range identified. ICD-9 codes were also used to ascertain specific mental disorder as follows: 295 for schizophrenia, 296 for bipolar disorder, 300 for anxiety, 301 for personality disorder, 311 for depressive disorder, 303 for alcohol dependence, and 304 for drug dependence.

### Availability of data and materials

Use of these data is governed by Information Sharing Agreements between the partnering ministries and the host academic research team. Access to data is subject to police security clearance, restricted to a designated secure off-line environment and other provisions to protect privacy. Additional details concerning these variables have been described elsewhere [34–37].

#### Baseline interview data

Baseline interview data were obtained by trained interviewers via in person administration of a detailed questionnaire consisting of several domains including: socio-demographics, physical health, mental and substance disorders and service use [30]. The following variables derived from the baseline self-report questionnaires were included in the multivariable analyses: gender, age, ethnicity (aboriginal; white; other). Aboriginal identity refers to the self-reported identification of being an Aboriginal person, that is, First Nations (North American Indian), Métis or Inuk (Inuit) [38]. In addition: level of education, lifetime duration of homelessness, age first homeless, multiple (3 or more) chronic health conditions, infectious disease (HIV, Hepatitis B or C), severity of mental disorder (severe or less severe), substance dependence, daily substance use, suicidality, and mental health severity were used to describe the socio-demographic characteristic of the sample. A “less severe” cluster of mental disorders included at least one of major depressive episode, post-traumatic stress disorder or panic disorder. A “severe” cluster included at least one of current psychosis, mood disorder with psychotic features and hypomanic or manic episode. Diagnostic information was based on the MINI 6.0 [30], with confirmation from a physician where available. No self-report diagnostic information was utilized in the multivariable analyses.

### Statistical analysis

We used descriptive statistics (mean and standard deviation for continuous variables; frequency and percentages for categorical variables) to characterize the study population. We used independent sample t tests to compare numerical variables (such as age at recruitment and homeless duration) and Pearson’s chi square test to compare categorical data (such as gender and ethnicity) between groups.

Due to the longitudinal nature of the data, we conducted Generalized Estimating Equations (GEE) analysis to estimate the correlations between outcome variables (acute hospital admissions and LOS) and predictor variables [39]. We selected negative binomial models (negative binomial regression; negative binomial distribution with log link) due to the over-dispersion and count nature of outcome data, and for better goodness of fit statistics relative to Poisson regression. Negative binomial distribution is widely used to analyze both hospital admission and LOS [40–42]. An autoregressive (first order) correlation structure and a robust method were chosen to control for dependency over time and to estimate standard errors for the parameters, respectively. We selected predictor variables a priori [5, 29] that were potentially associated with hospital admissions and LOS and analyzed them as time-varying covariates (measured at each pre-recruitment year) in the GEE regression models. As predictors, we have chosen schizophrenia, bipolar disorder, personality disorder, depressive disorder and neurotic/anxiety disorder as non substance-related mental disorders; and drug dependence and alcohol dependence as substance use disorders (derived from administrative data).

We examined the effects of specific mental disorders on outcome variables in both bivariate and multivariable analyses settings. We selected age (measured at each pre-recruitment year), gender (male and female), ethnicity (white, aboriginals), time, and non-psychiatric service utilization (services for non-psychiatric diagnoses including medical and paramedical services, as paid for by MSP) as potential confounders and forced them in multivariable models regardless of their significance in bivariate setting. Age, gender and ethnicity were derived from self-report data.

We reported rate ratios along with 95 % confidence intervals as measures of association (effect sizes). We chose the conventional alpha level (*p* ≤0.05) to report significance for the estimated parameters. All reported p-values were two sided. Participants with missing responses were excluded from the analysis.

IBM SPSS Statistics 22 (Ref: IBM Corp. Released 2013. IBM SPSS Statistics for Windows, Version 22.0. Armonk, NY: IBM Corp.) and STATA 13 (Ref: StataCorp. 2013. *Stata Statistical Software: Release 13*. College Station, TX: StataCorp LP) were used to conduct these analyses.

## Results

The characteristics of participants who consented to accessing administrative data and whose records could be matched (*n* = 433) were compared to the full sample (*n* = 497) on a number of socio-demographic characteristics (see Table 1). The overall pattern of findings indicated the eligible sample did not differ significantly from the larger cohort. Members of the eligible sample were predominantly male (73.9 %), self identified as Caucasian (54.3 %), with a mean age of 40.8 years. Seventy - two percent of participants met diagnostic criteria for the “severe” cluster of mental disorders, either schizophrenia, or bipolar disorder, and over half (58.2 %) met criteria for current substance dependence.

**Table 1:** Socio-demographic characteristics of ‘At home’ participants by consent status at enrolment visit

Variable	The entire sample (*n* = 497) n (%)/mean (SD)^c^	Eligible sample^a^ (*n* = 433) n (%)/mean (SD)	Not eligible sample^b^ (*n* = 64) n (%)/mean (SD)	*P* value^*^
Age at randomization (in years)	40.8 (11.0)^c^	40.8 (11.0)^c^	41.4 (11.0)^c^	0.682
Age of first homelessness (in years)	30.3 (13.3)^c^	30.1 (13.4)^c^	31.9 (12.6)^c^	0.301
Female gender	134 (27.2)	112 (26.1)	22 (34.4)	0.165
Ethnicity				
Aboriginals	77 (15.5)	70 (16.2)	7 (10.9)	0.054
White	280 (56.3)	235 (54.3)	45 (70.3)	
Other	140 (28.2)	128 (29.6)	12 (18.8)	
Incomplete High School	280 (56.7)	247 (57.4)	33 (51.6)	0.376
Single/Never Married	343 (69.6)	293 (68.1)	50 (79.4)	0.071
Need level (high)	297 (59.8)	255 (58.9)	42 (65.6)	0.305
Housing first interventions	297 (59.8)	257 (59.4)	40 (62.5)	0.632
Lifetime duration of homelessness (in months)	60.2 (70.3)^c^	58.3 (64.8)^c^	72.9 (99.8)^c^	0.124
Longest episode of homelessness (in months)	30.9 (40.1)^c^	30.4 (39.5)^c^	34.1 (44.4)^c^	0.498
Less severe cluster of mental disorders	264 (53.1)	235 (54.3)	29 (45.3)	0.180
Severe cluster of mental disorders	363 (73.0)	311 (71.8)	52 (81.3)	0.113
Suicidality (high)	87 (17.5)	79 (18.2)	8 (12.5)	0.259
Substance dependence	288 (57.9)	252 (58.2)	36 (56.3)	0.768
Daily substance use	143 (28.9)	131 (30.4)	12 (19.0)	0.064
Mental health severity/CSI score (per unit)	37.2 (12.5)^c^	37.4 (12.5)^c^	35.8 (12.9)^c^	0.371
Chronic Medical Conditions (3 or more)	344 (69.2)	305 (70.4)	39 (60.9)	0.124
Blood-borne Infectious Disease (HIV, Hepatitis B or C)	157 (31.9)	139 (32.3)	18 (29.0)	0.603

^a^-Out of 497 participants, 433 provided consent to access to administrative health data and were linkable to health records

^b^-Out of 64 participants, 60 didn’t consent to access to administrative health data and four provided consent, but were unlinkable to health records

^c^-Indicates mean (SD)

^*^-*P* values based on comparisons of characteristics between eligible participants and non-eligible participants in the entire sample

**Fig. 1:**
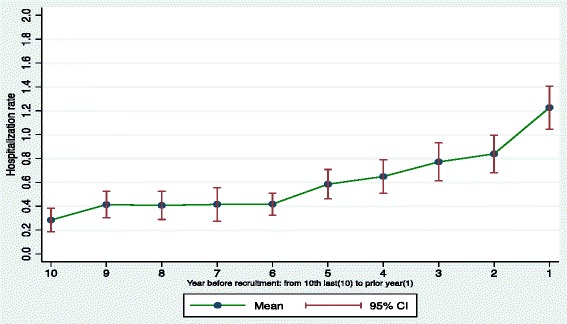
Acute hospitalization before recruitment for ‘At Home’ participants (*n* = 433)

**Fig. 2:**
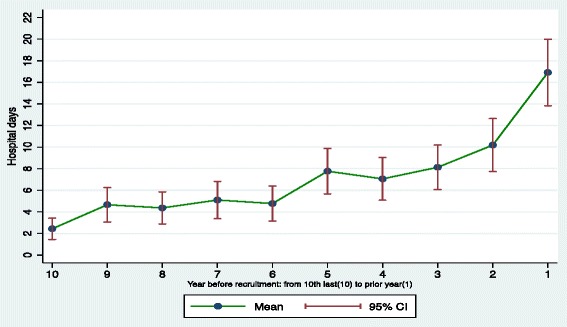
Hospital days before recruitment for ‘At Home’ participants (*n* = 433)

Table 2 displays the annual hospitalization rates and LOS for the sample over a 10-year pre-recruitment period. During the observation period, participants (*n* = 433) had on average, 6.0 inpatient admissions per person, with a total of 2601 hospitalizations. Of the 2601 acute hospital admissions, 1982 were psychiatric and 619 were non-psychiatric hospitalizations. Mean rates for annual hospital admission increased from 0.3 to 1.2 (per person per year), between the 10th year and 1st year prior to recruitment (see Fig. 1). The average cumulative LOS was 71.4 days per person over the study period (10 years), with annual mean rates increasing from 2.4 to 16.9 (per person per year) between the 10th and 1st year prior to recruitment (see Fig. 2). Eighty percent of the sample (*n* = 342) accounted for the total number of psychiatric and non-psychiatric hospitalizations.

**Table 2:** Annual hospitalization rate (from 1st year to 10th year) for ‘At Home’ participants (*n* = 433) during pre-recruitment period

Service area	10th	9th	8th	7th	6th	5th	4th	3rd	2nd	1st	Entire period (10-years)
Acute hospital admissions Mean (SD)	0.3 (1.0)	0.4 (1.2)	0.4 (1.2)	0.4 (1.5)	0.4 (1.0)	0.6 (1.3)	0.6 (1.5)	0.8 (1.7)	0.8 (1.7)	1.2 (1.9)	6.0 (8.6)
Non-psychiatric hospital admissions	0.1 (0.4)	0.1 (0.3)	0.1 (0.5)	0.1 (0.4)	0.1 (0.4)	0.2 (0.5)	0.2 (0.5)	0.2 (1.1)	0.2 (0.6)	0.2 (0.7)	1.4 (2.4)
Psychiatric^a^ hospital admissions	0.2 (1.0)	0.3 (1.1)	0.3 (1.1)	0.3 (1.4)	0.3 (0.9)	0.4 (1.2)	0.5 (1.4)	0.5 (1.3)	0.7 (1.5)	1.0 (1.8)	4.6 (8.1)
Length of stay Mean (SD)	2.4 (10.5)	4.7 (16.9)	4.4 (15.7)	5.1 (18.4)	4.8 (17.2)	7.8 (22.3)	7.1 (20.9)	8.1 (21.8)	10.2 (26)	16.9 (32.5)	71.4 (123.3)
Any hospitalization N (%)	54 (12.5)	77 (17.8)	76 (17.6)	75 (17.3)	97 (22.4)	120 (27.7)	118 (27.3)	147 (33.9)	153 (35.3)	206 (47.6)	342 (79.0)
Tertiary psychiatric hospitalization^b^ N (%)	0 (0.0)	4 (0.9)	6 (1.4)	8 (1.8)	7 (1.6)	9 (2.1)	5 (1.2)	6 (1.4)	8 (1.8)	13 (3.0)	41 (9.5)

^a^-Includes either non-substance related or substance related mental disorders

^b^-Facility name is Riverview Hospital

The effects of specific mental disorders were examined in relation to hospitalization rates (Table 3) and LOS (Table 4) during a 10-year pre-recruitment period. Unadjusted (URR) and adjusted rate ratios (ARR) are provided with 95 % confidence intervals (CI). In unadjusted and adjusted analyses all psychiatric diagnostic categories, as well as drug dependence, were significantly (*p* <0.001) associated with hospital admissions (Table 3), accompanied by varying magnitudes of effect [43]. When compared to individual’s not meeting criteria for either disorder independently, participants with a diagnosis of schizophrenia [ARR 4.7 (3.9, 5.6)] or bipolar disorder [ARR 2.2 (1.8, 2.6)] demonstrated strong and moderate associations with hospital admission. Weak to moderate associations with hospital admission were observed among participants with: personality disorder [ARR 1.6 (1.8, 2.6)], depressive disorder [ARR 1.6 (1.3, 1.9)], anxiety-neurotic disorder [ARR 1.5 (1.2, 1.8)], and drug dependence [ARR 1.5 (1.2, 1.7)], when compared to individuals without these disorders. In addition, high (≥32) non-psychiatric service utilization [ARR 2.3 (1.9, 2.8)] was significantly (*p* <0.001) associated with acute hospitalization, when compared to low (≤15) non-psychiatric service use.

**Table 3:** GEE regression analysis to estimate the effects of specific mental disorders on hospitalizations for ‘At Home’ participants during a 10-year pre-recruitment period (*n* = 433)

Independent variables	Unadjusted Rate Ratio (95 % CI)^a^	*P* value	Adjusted Rate Ratio (95 % CI)^b^	*P* value
Age at each pre-recruitment year (per year)	1.0 (1.0. 1.0)	0.048	1.0 (1.0, 1.0)	0.012
Time (per year)	1.2 (1.1. 1.2)	<0.001	1.1 (1.0, 1.1)	<0.001
Female gender	1.1 (0.9, 1.5)	0.349	1.0 (0.8, 1.2)	0.813
Aboriginals (yes vs. no)	0.9 (0.7, 1.3)	0.635	1.1 (0.8, 1.4)	0.617
White (yes vs. no)	1.1 (0.8, 1.4)	0.594	0.9 (0.7, 1.1)	0.217
Schizophrenia (yes vs. no)	7.7 (6.4, 9.3)	<0.001	4.7 (3.9, 5.6)	<0.001
Bipolar disorder (yes vs. no)	5.7 (4.6, 7.0)	<0.001	2.2 (1.8, 2.6)	<0.001
Personality disorder (yes vs. no)	3.0 (2.3, 3.9)	<0.001	1.6 (1.8, 2.6)	<0.001
Depressive disorder (yes vs. no)	3.2 (2.7, 3.8)	<0.001	1.6 (1.3, 1.9)	<0.001
Anxiety-neurotic disorder (yes vs. no)	2.8 (2.3, 3.5)	<0.001	1.5 (1.2, 1.8)	<0.001
Alcohol dependence (yes vs. no)	2.5 (1.9, 3.2)	<0.001	1.5 (1.2, 2.0)	0.001
Drug dependence (yes vs. no)	2.4 (2.0, 2.9)	<0.001	1.5 (1.2, 1.7)	<0.001
Non-psychiatric service utilization^c^				
High (≥32)	3.4 (2.7, 4.3)	<0.001	2.3 (1.9, 2.8)	<0.001
Medium (16 to 31)	2.1 (1.7, 2.7)	<0.001	1.5 (1.2, 1.8)	<0.001
Low (≤15)	Reference	-	Reference	-

^a^-The Confidence Intervals were estimated using Robust Standard errors

^b^- The Confidence Intervals were estimated using Robust Standard errors

^c^-50th and 75th percentile were used as a cutoff value for medium and high groups respectively

**Table 4:** GEE regression analysis to estimate the effects of specific mental disorders on length of stay for ‘At Home’ participants during a 10-year pre-recruitment period (*n* = 433)

Independent variables	Unadjusted rate ratio (95 % CI)^a^	*P* value	Adjusted rate ratio (95 % CI)^b^	*P* value
Age at each pre-recruitment year (per year)	1.0 (1.0. 1.0)	0.012	1.0 (1.0, 1.0)	0.314
Time (per year)	1.2 (1.2. 1.2)	<0.001	1.1 (1.0, 1.1)	0.002
Female gender	1.3 (0.9, 1.8)	0.182	1.1 (0.8, 1.6)	0.427
Aboriginals (yes vs. no)	0.7 (0.5, 1.1)	0.103	0.8 (0.5, 1.2)	0.200
White (yes vs. no)	1.0 (0.8, 1.4)	0.877	0.9 (0.6, 1.2)	0.426
Schizophrenia (yes vs. no)	15.6 (12.0, 20.4)	<0.001	13.7 (9.7, 19.5)	<0.001
Bipolar disorder (yes vs. no)	7.6 (5.9, 9.8)	<0.001	3.5 (2.4, 5.0)	<0.001
Personality disorder (yes vs. no)	2.2 (1.5, 3.2)	<0.001	1.6 (1.0, 2.5)	0.069
Depressive disorder (yes vs. no)	2.8 (2.2, 3.6)	<0.001	1.7 (1.2, 2.3)	0.003
Anxiety-neurotic disorder (yes vs. no)	2.4 (1.9, 3.0)	<0.001	1.4 (1.0, 1.9)	0.034
Alcohol dependence (yes vs. no)	1.9 (1.3, 2.6)	<0.001	1.4 (0.9, 2.3)	0.185
Drug dependence (yes vs. no)	1.8 (1.4, 2.2)	<0.001	1.6 (1.1, 2.2)	0.012
Non-psychiatric service utilization^c^				
High (≥32)	2.4 (1.8, 3.1)	<0.001	3.4 (2.2, 5.2)	<0.001
Medium (16 to 31)	1.5 (1.1, 2.1)	0.014	1.2 (0.8, 1.8)	0.286
Low (≤15)	Reference	-	Reference	-

^a^-The CI were estimated using Robust Standard errors

^b^- The CI were estimated using Robust Standard errors

^c^-50th and 75th percentile were used as a cutoff value for medium and high groups respectively

Table 4 estimates the effects of specific mental disorders on LOS. In adjusted analyses, diagnoses of schizophrenia [ARR 13.7 (9.7, 19.5)] or bipolar disorder [ARR 3.5 (2.4, 5.0)] were strongly associated with longer hospitalizations, accounting for 14 and 3.5 more days, respectively, in comparison to individuals without these disorders. Depressive disorder [ARR 1.7 (1.2, 2.3)], and drug dependence [ARR 1.6 (1.1, 2.2)] had a moderately significant (*p* < 0.05) association with LOS in comparison to the absence of these disorders. Moreover, high (≥32) non-psychiatric service utilization (number of services for non-psychiatric diagnoses including medical and paramedical services, as paid for by MSP) was independently associated with nearly 3.5 [ARR 3.4 (2.2, 5.2)] more hospital days, when compared to low (≤15) non-psychiatric service utilization.

## Discussion

Our findings reveal that specific mental disorders and high frequency non-psychiatric service use were significantly associated with acute hospitalization and longer lengths of stay. To our knowledge this is the first study examining the associations between specific mental illnesses and hospital admissions and LOS in a cohort of homeless people with mental illness spanning a decade. The use of 10-year longitudinal data provides a robust measure, enhancing detection of changes and patterns in both diagnostic and health service utilization across a subpopulation of individuals known to be associated with high levels of service use. These results demonstrate specific diagnostic risk factors associated with hospital admission and LOS, with implications for tertiary prevention, including the provision of housing and supports to mitigate medical emergencies and acute service use.

We found that 80 % of participants were admitted to hospital over the course of the study, corresponding to a mean rate of 1.4 admissions per person for non-psychiatric hospitalization and 4.6 admissions per person for psychiatric hospitalizations. The number of admissions to hospital progressively climbed representing a four-fold increase over the 10-year study period (see Fig. 1). Our results are higher than previously reported rates of hospitalizations among homeless individuals [4, 23, 44] and are considerably above the annual age-standardized acute inpatient hospitalization rates for the general population of BC and Canada (Canadian Institute for Health Information, 2013). Differences in study duration, geographic location, and health insurance status may contribute to these discrepancies in hospitalization rates within the literature. More importantly, this variation reflects the need for further examination of homeless populations and the sources of heterogeneity in acute health services use. Psychiatric admissions accounted for 76.2 % of all admissions to hospital in our sample. This finding differs from the literature in which medical service visits accounted for a higher proportion [1, 5, 44], with other studies showing comparable percentages [4]. This result may reflect the severity of psychiatric symptoms and co-occurring substance use in our sample.

Our results indicate that participants with more chronic psychiatric disorders, such as schizophrenia and bipolar disorder were associated with significantly higher rates of hospital admission and longer stays once admitted, when compared to individuals diagnosed with other mental disorders. Similar results have been reported in general population samples [29]; however, few studies have replicated this finding among the homeless. In adjusted and unadjusted models, a diagnosis of schizophrenia was associated with 4.5 times the rate of admissions and 14 more days spent in hospital when compared to other mental and substance use disorders. Previous research has shown that mental disorders, when considered as a class and regardless of severity or symptomatology, are predictive of health service use among homeless populations [7, 23, 44]. In addition, prior studies indicate that clinical characteristics and severity of symptoms (i.e. presence of delusions) alongside lower levels of psychosocial functioning are associated with increased admission to hospital [29, 45], aligning with results demonstrated in our study. Our study extends these earlier findings by demonstrating the utility of disaggregating specific mental disorders as possible predictors of hospitalization. Furthermore, use of diagnostic categories as defined by the ICD-9, an international classification system, strengthens the widespread ability to examine other chronically homeless populations with similar diagnostic classifications in an effort to guide interventions or improve service use outcomes.

Diagnoses of alcohol or drug dependence were not strong predictors of hospitalization or LOS, despite prior studies demonstrating associations with other types of acute health service use, such as emergency departments [1, 2, 7, 46]. This finding was contrary to previous studies involving both homeless [4, 21, 22] and non-homeless individuals [47], which reported links between substance dependence and admission to hospital. In addition, prior research suggests an increased risk for admission among patients with co-occurring disorders, (i.e., presence of substance dependence and mental disorder) [4, 47]. This contradictory finding is likely a result of several factors, such as clinical presentation, patient safety, and ability to provide effective treatment in an emergency department setting versus inpatient bed. The provision of treatment for substance related issues, via emergency departments’ or residential treatment programs limits the need for inpatient care, however it does not necessarily reduce healthcare costs. Further research is needed to investigate how substance use impacts the use of inpatient services.

Nearly 20 % of participants did not account for any hospital contact during the observation period. The absence of hospitalization among this subgroup is noteworthy, given the high prevalence of substance use disorders and multiple medical conditions as well as mental illness in the sample. Despite a self-reported need for care [16], barriers including stigma have resulted in homeless people receiving or accessing inadequate medical care in the community [17, 18]. These barriers, and the generally insufficient availability of care for the homeless results in health conditions going untreated or, alternatively, frequent use of ‘non-discretionary services’ [4, 28]. Our results also indicate that high non-psychiatric service use (i.e., physician visits, laboratory and/or diagnostic investigations, etc.) was associated with increased rates of admissions and longer stays once admitted. This result supports the importance of comprehensive care planning, in particular among populations with markers of medical comorbidity, whereby medical and psychiatric care encounters are jointly acknowledged as key indicators of appropriate patient care and health policy planning.

We observed an annual increase in the mean LOS over the study period (see Fig. 2). Participants averaged 71.4 days per person in hospital over 10 years, representing nearly 31,000 days spent hospitalized. This large number of days signifies intensive health resource utilization that cannot solely be attributed to homeless status or mental disorders; but nevertheless contributes to substantial health care costs for homeless individuals when compared to stably housed patients [4, 26]. Omissions in discharge planning, such as lack of housing status evaluation while hospitalized, have been associated with increased days spent hospitalized among the homeless [48]. In turn, readmission rates have been used as indicators of appropriate discharge planning and care quality [49]. Homeless individuals are linked to high rates of re-admission within the 30-day period following index hospitalization [26, 27] or ED visit [50]. Although we did not directly measure re-admission rates, schizophrenia and psychotic disorders were predictive of readmission as reported by Mark et al. [49] and these same disorders were associated with increased LOS in our analyses.

Limitations to this study include the fact that administrative records related to hospitalization and LOS were restricted to individuals with PHN’s from the province of BC and therefore do not include care received outside BC. Our sample was predominantly white and male, and our research took place in a context of universally available publicly funded healthcare. These factors may limit the generalizability of our findings to other contexts. Finally, people who met eligibility criteria and consented to enroll in the study may be systematically different than individuals who declined to participate or were excluded.

Despite the limitations reported here, this study has noteworthy strengths, including the use of comprehensive administrative health data with health service contact records spanning a 10-year period. The addition of disaggregated data alongside extensive health record linkage adds novel information to the existing literature concerning chronically homeless adults with mental and substance use disorders. Additionally, the study sample met stringent criteria for inclusion representing homelessness and mental illness. The findings reported here comprise a step toward better understanding of the long-term associations between hospitalization and diagnostic variables among people experiencing prolonged homelessness.

## Conclusion

Our results demonstrate that the presence of chronic psychiatric disorders as well as high rates of non-psychiatric health service use are strong predictors of acute hospital admission and LOS. Further research and examination of the risk factors that place particular homeless and mentally ill individuals at risk for acute medical crises is recommended in conjunction with improved detection and early intervention. Early detection and screening are important since homelessness creates a preventable burden on individual health and health care resources. Further research and interventions must be tailored towards homeless individuals with more severe mental disorders and documented high service use in order to lessen the extreme pressure currently placed on individuals as well as on community resources. Hospitalization rates and LOS are important indicators of health service performance, and policy leaders need to respond accordingly.

## Abbreviations

ARR: Adjusted rate ratio
BC: British Columbia
CI: Confidence intervals
ED: Emergency department
GEE: Generalized Estimating Equation
ICD: International classification of disease
LOS: Length of stay
MSP: Medical services plan
NSMD: Non substance-related mental disorders
PHN: Personal health number
SUD: Substance use disorders
URR: Unadjusted rate ratio
